# An ensemble-averaged, cell density-based digital model of zebrafish embryo development derived from light-sheet microscopy data with single-cell resolution

**DOI:** 10.1038/srep08601

**Published:** 2015-02-25

**Authors:** Andrei Y. Kobitski, Jens C. Otte, Masanari Takamiya, Benjamin Schäfer, Jonas Mertes, Johannes Stegmaier, Sepand Rastegar, Francesca Rindone, Volker Hartmann, Rainer Stotzka, Ariel García, Jos van Wezel, Ralf Mikut, Uwe Strähle, G. Ulrich Nienhaus

**Affiliations:** 1 Institute of Applied Physics, Karlsruhe Institute of Technology, Wolfgang-Gaede-Str. 1, 76131 Karlsruhe, Germany; 2 Institute of Toxicology and Genetics, Karlsruhe Institute of Technology, Post Office Box 3640, 76021 Karlsruhe, Germany; 3 European Zebrafish Resource Centre, Karlsruhe Institute of Technology, Post Office Box 3640, 76021 Karlsruhe, Germany; 4 Institute for Applied Computer Science, Karlsruhe Institute of Technology, Post Office Box 3640, 76021 Karlsruhe, Germany; 5 Institute for Data Processing and Electronics, Karlsruhe Institute of Technology, Post Office Box 3640, 76021 Karlsruhe, Germany; 6 Steinbuch Centre for Computing, Karlsruhe Institute of Technology, Post Office Box 3640, 76021 Karlsruhe, Germany; 7 Department of Physics, University of Illinois at Urbana-Champaign, Urbana, Illinois 61801, USA

## Abstract

A new era in developmental biology has been ushered in by recent advances in the quantitative imaging of all-cell morphogenesis in living organisms. Here we have developed a light-sheet fluorescence microscopy-based framework with single-cell resolution for identification and characterization of subtle phenotypical changes of millimeter-sized organisms. Such a comparative study requires analyses of entire ensembles to be able to distinguish sample-to-sample variations from definitive phenotypical changes. We present a kinetic digital model of zebrafish embryos up to 16 h of development. The model is based on the precise overlay and averaging of data taken on multiple individuals and describes the cell density and its migration direction at every point in time. Quantitative metrics for multi-sample comparative studies have been introduced to analyze developmental variations within the ensemble. The digital model may serve as a canvas on which the behavior of cellular subpopulations can be studied. As an example, we have investigated cellular rearrangements during germ layer formation at the onset of gastrulation. A comparison of the *one-eyed pinhead* (*oep*) mutant with the digital model of the wild-type embryo reveals its abnormal development at the onset of gastrulation, many hours before changes are obvious to the eye.

Zebrafish (*Danio rerio*) is widely used as a model organism in developmental and biomedical research. The high conservation of genes^1^ provides an opportunity to explore mechanisms of a wide range of human pathologies using the zebrafish. Currently, the number of zebrafish mutants is rapidly increasing due to large-scale screening efforts^2^ as well as new technology for site-directed mutagenesis^3^. Soon, there will be at least one mutation introduced in each of the 26,206 protein coding genes^1^ of the zebrafish genome. The number of transgenic lines marking various cellular structures and processes in the developing embryo by fluorescent protein tags is also swiftly growing. This vast number of zebrafish lines poses severe challenges to stock maintenance and archiving of phenotypes. They will have to be preserved as frozen sperm, and content-rich documentation methods should enable researchers to pre-screen mutant phenotypes and transgene expression patterns prior to reviving a particular line.

Optical fluorescence microscopy is arguably the most powerful and content-rich method to investigate entire millimeter-sized organisms such as zebrafish embryos. Images can be taken with submicron resolution by using confocal microscopy^4^, light-sheet microscopy (LSM)^5^ or optical projection tomography (OPT)^6^. Among these, LSM is especially attractive for whole-organism imaging because its side-on excitation in the form of a thin light sheet minimizes photobleaching and phototoxicity. LSM affords high-speed imaging over long periods of time with single-cell spatial resolution and enables a detailed reconstruction of morphological dynamics^5^,^7^,^8^,^9^,^10^,^11^,^12^,^13^,^14^. An important LSM application is large-scale screening of sometimes subtle phenotypical changes of embryos induced by mutations or environmental conditions. A key requirement in this work is an accurate, four-dimensional (4D, three spatial dimensions plus time) digital model of an organism's normal development as a reference. Such a model must be based on multi-sample averaging to remove variations between individual embryos from the same experimental group.

Quantitative 4D imaging is extremely challenging because vast amounts of data (~1 TB/h) are generated that need to be handled and analyzed efficiently. An obvious strategy to alleviate this problem is rigorous data reduction at the time of acquisition, based on the specific shape of the sample. For example, in zebrafish embryos, cells are distributed on top of the yolk sphere at early developmental stages. Consequently, the cuboid-shaped observation volume of an LSM contains a large number of volume elements (voxels) that do not carry any sample information. A geometric transformation from a Cartesian to a spherical coordinate system allows only those voxels to be stored that contain sample information, which drastically reduces the overall size of the data set^15^. However, this method is not applicable to samples of arbitrary shape and, moreover, the raw data are discarded and cannot be reprocessed at a later time.

Here we present a large-scale data acquisition and analysis framework for light-sheet microscopy recording with an optimized data flow that preserves all raw image information. It is based on a home-built digital scanned laser light sheet microscope (DSLM)^5^ that is capable of high-speed 3D image acquisition over many hours with single cell resolution. To process the enormous flow of imaging data efficiently, we have developed semi-automated tools for multi-sample averaging and introduced quantitative metrics for the analysis of embryo morphological dynamics. To reveal “normal” zebrafish morphogenesis during the first 16 h of development, we have imaged an ensemble of zebrafish embryos with fluorescently labeled nuclei under identical conditions. From these data, an ensemble-averaged digital embryo model (available as Matlab file at http://www.aph.kit.edu/nienhaus/english/26_409.php#DigitalEmbryoModel; the structure of the file is described in Supplementary Table 1) was developed that can serve as a reference for samples showing “abnormal” behavior. This digital model represents a 4D (3D + time) array of cell densities with a ~10-μm grid size and a 4D (vector) array of cell movement directions that allows reconstruction of local pattern formation on a scale of a few tens of micrometers. The digital model can be used as a reference frame, or “canvas”, for studying subpopulations of cells that can be identified in the images, e.g., by their specific expression of fluorescent tags or their peculiar dynamics, as shown here for hypoblast and epiblast cells. Furthermore, by comparing data on the *one-eyed pinhead* (*oep*) zebrafish mutant with our model, we observe morphological changes already at the early gastrula stage, many hours before morphological abnormalities are visible by eye.

## Results

### Acquisition of large-scale DSLM data sets

The ultimate goal of developmental biology is to establish the complete lineage of each cell within an organism^16^,^17^. To be able to unambiguously track individual cells, their displacement from one image to the next must be less than half their separation from adjacent cells at all times during many hours of development. At present, complete cell lineage reconstruction is still challenging due to incomplete cell segmentation in areas with reduced image quality and tracking errors in regions with high cell density, however^12^. With our home-built DSLM, we measure complete 3D images (two opposite views, each consisting of 500 frames with 2 μm spacing in depth) every 50 s; even faster acquisition speeds can be achieved by restricting the depth and the field of view (for details, see Methods and Supplementary Figs. 1 and 2).

**Figure 1:**
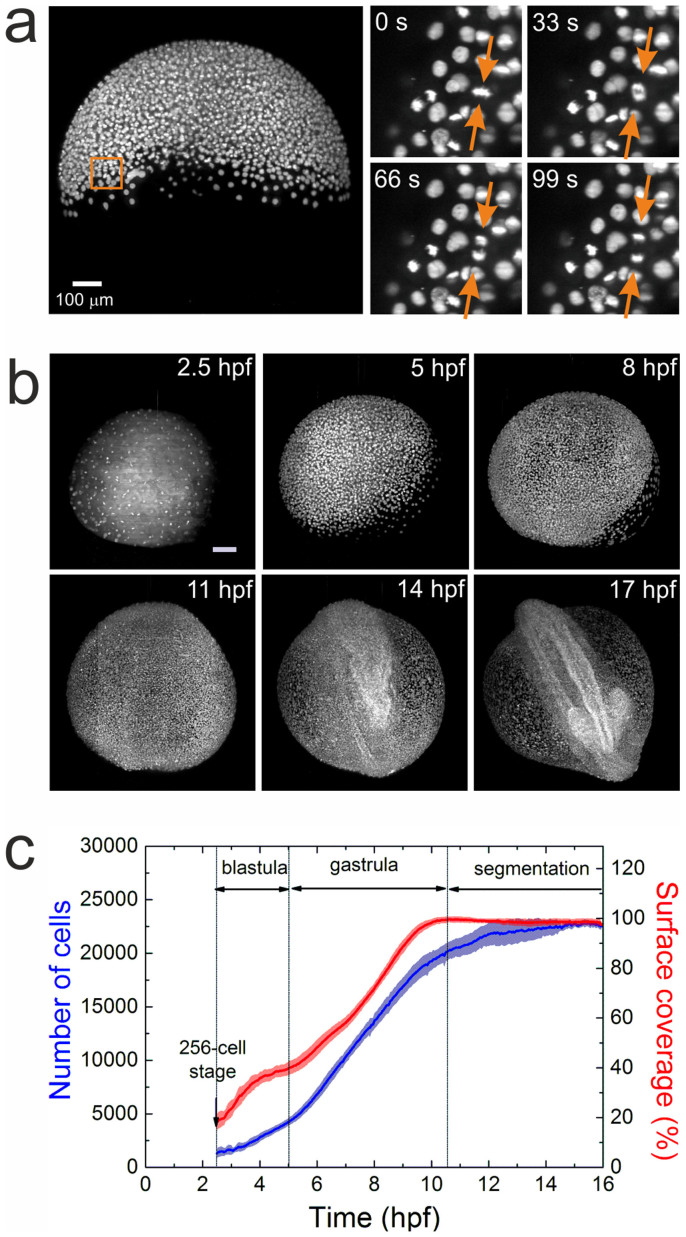
Imaging and quantification of embryo development. (a) MIP image of an embryo at 50% epiboly; a sequence of four time-lapse images from a selected region (square) is shown on the right. The arrows point to a dividing cell. (b) Typical maximum intensity projection images of a zebrafish embryo at 2.5, 5, 8, 11, 14, and 17 hpf. Scale bar is 100 μm. (c) Time dependence of the average cell number and yolk surface coverage during embryo development, computed from an ensemble of five embryos.

For imaging, dechorionated zebrafish embryos were mounted in vertically oriented FEP tubes, in which they sat on a plug of 1.5% agarose. The embryos were surrounded by only 0.1% agarose to minimize frictional effects on the moving cells (Supplementary Fig. 1c). Fig. 1a displays a maximum intensity projection (MIP) image at ~50% epiboly. A set of four consecutive images from a selected region of 100 × 100 × 20 μm^3^ at the blastoderm margin clearly shows that cell division can be reliably detected under these conditions; most cells can be tracked for several hours. Typical MIP images of a zebrafish embryo at selected time points are displayed in Fig. 1b. Data for the digital embryo model were collected on ten cell nuclei-stained zebrafish embryos. They were imaged over 16 h, starting from the cleavage periods of the 8–64 cell stages (Supplementary Movie 1). Five data sets showing normal embryo development, i.e., head, somites and tail formation at 24 hours post fertilization (hpf)^18^, were selected by visual inspection for further segmentation analysis (for details, see Supplementary Materials and Methods). To synchronize the developmental time axis for different samples, we selected the 256-cell stage as the starting point, corresponding to 2.5 hpf. At this time, the fluorescence from the nuclei became predominant, allowing an unambiguous tracking of the cells. During the following 14 h of measurement, blastula (2.5–5 hpf), gastrula (5–10.5 hpf) and somite segmentation (10.5–16.5 hpf) stages of development were recorded and analyzed. The time dependencies of the average cell number and the yolk surface coverage with cells are displayed in Fig. 1c for the selected zebrafish ensemble. Evidently, sample-to-sample variations are small and do not exceed 10% within the observed time window. The different developmental stages can be identified from the rate of change of the average cell number (Fig. 1c, blue). After a moderate increase during the blastula stage, the rate increases in the gastrula stage and slows again during the somite segmentation period. The cell nuclei segmentation quality may be reduced at later times due to the high cell density, which may also contribute to the smaller cell proliferation rate observed beyond 12 hpf. We have also estimated the yolk surface coverage (Fig. 1c, red) by calculating the area that the cells (approximated by spheres of 25 μm in diameter) occupy on the yolk sphere (typical diameter 650–700 μm). Complete coverage defining the end of the gastrula stage is followed by a slight decrease during segmentation due to reduced cell density at the ventral side of the embryo. Notably, a slight undulation is observed in the monotonic increase of yolk surface coverage during gastrulation between 5 and 7 hpf, which is likely related to germ ring formation, an inward cell movement from the blastodermal to the inner hypoblast layer^19^,^20^.

### Sample alignment to a standard orientation

**Figure 2:**
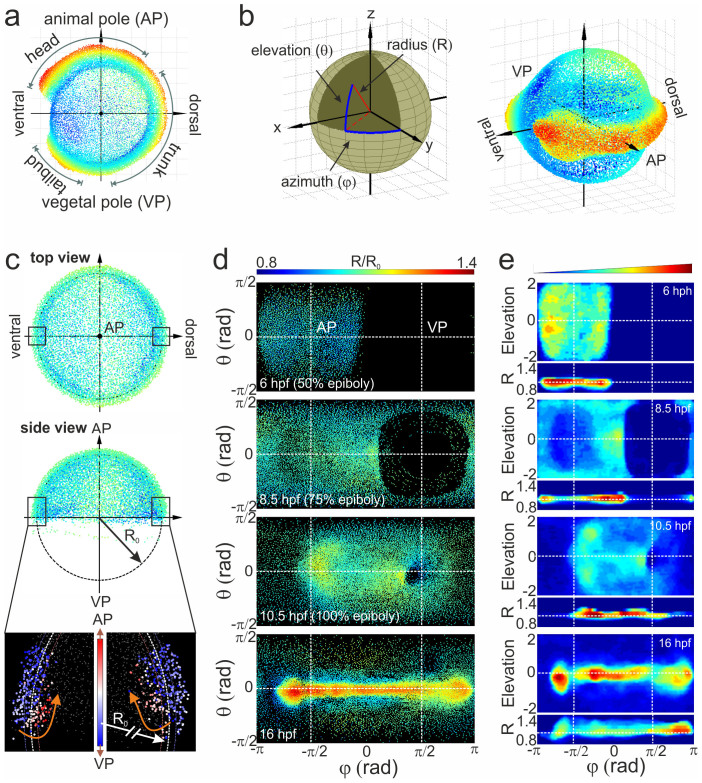
Alignment of cell coordinates according to the standard embryo position. (a) Standard embryo representation on the yolk sphere at later developmental time points (16 hpf); the color code denotes the distance from the origin. (b) Transformation from Cartesian to spherical coordinates. For the cylindrical projection, the anterior-posterior embryo axis was placed at zero elevation (equator); by setting the dorsal embryo side at zero azimuths, the animal (AP) and vegetal (VP) poles appeared at the −π/2 and π/2 azimuthal angles, respectively. (c) Embryo alignment at the early gastrula stage. Top and side views show the symmetrically distribution of cells around the AP. The color code denotes the distance from the origin as in (a). The radius of the embryo sphere R_0_ is determined according to the border between hypoblast and epiblast cell layers (lower panel of the side view; the color code denotes the movement direction) which display opposite movement at 7 hpf towards the AP (red) and VP (blue), respectively. (d) Spherical projection of cell nuclei coordinates from a single embryo on a 2D map using azimuthal and elevation angles. Typical 2D maps of cell nuclei coordinates are plotted at 6, 8.5, 10.5 and 16 hpf using the equidistant cylindrical projection. The radial coordinate is color-coded in the range between 0.8 and 1.4. (e) 2D maps of integrated cell nuclei density from a single sample using the area-preserving Gall-Peters (2 sinθ versus φ) and R versus φ projection of the same datasets as in (d). The blue-to-red color code denotes the increase of the normalized cell density in arbitrary units.

Careful alignment of cell distributions from each sample at each point in time is of utmost importance for construction of the digital embryo model as well as for quantitative comparison of embryo morphogenesis between multiple samples. We chose the 3D representation depicted in Fig. 2a, with the animal and vegetal poles (AP and VP) on the top and bottom, and the ventral and dorsal sides on the left and right, respectively, as the standard orientation. At early developmental stages, cells are distributed mainly as a thin layer on top of the yolk sphere and, therefore, the projection of 3D image data onto a sphere and its unwrapping as a 2D map enables visualization of all relevant embryo features^15^. In Fig. 2b, the transformation from Cartesian to spherical coordinates and the spatial orientation of an embryo using azimuthal and elevation angles and radial distance are shown. To account for sample-to-sample variations of the embryo radius (330 μm on average), we normalize the radius of the sphere separating hypoblast and epiblast cell layers to unity. These cells can be distinguished by their movement direction at 7 hpf^5^. Thus, cells showing internalization and movement away from the blastoderm margin are assigned to hypoblast cells, whereas cells moving towards the VP with the blastoderm margin are assigned to epiblast cells (Fig. 2c). Furthermore, the epiboly percentage can be quantitatively calculated from the aligned cell distribution by taking the margin height along the AP-VP axis. Its comparison with the surface coverage presented in Supplementary Fig. 3 reveals the same time pattern that is delayed by 5–10% due to excluded yolk syncytial layer cells from the margin height evaluation. Different methods of spherical data projections exist, preserving one or another parameter^21^. We use the equidistant (plot of elevation angle, θ, versus azimuthal angle, φ, Fig. 2d) and the area-preserving Gall-Peters cylindrical projections (2 sinθ versus φ, Fig. 2e) for visualizing cell location and density, respectively. The equidistant cylindrical projection induces substantial distance and angle distortions at the edges of the elevation axis; however, it affords the simplest relation between nuclei positions on the map and on the embryo sphere. The Gall-Peters projections likewise distorts distances and angles, but reproduces different features (e. g., brain rudiment and eye) along the elevation axis properly scaled.

To transform the cell nuclei coordinates from camera pixel/frame coordinates to aligned spherical coordinates for each point in time, we developed an automated algorithm that fits the sphere into real-space cell coordinates and then rotates the sphere such that every sample is oriented according to the following rules: (i) cells are symmetrically distributed with respect to the midline; (ii) for time points beyond 50% epiboly, the elevation center of gravity of cells at different azimuthal angles lies on the equator line (corresponding to the future anterior-posterior body axis), (iii) for times before 50% surface coverage, cells are symmetrically distributed with respect to the −π/2 azimuthal angle (corresponding to the AP), (iv) for times between 50% and 100% epiboly, the center of azimuthal symmetry is linearly shifted between −π/2 and 0 azimuthal angles, and (v) for times beyond 100% surface coverage, cells are symmetrically distributed with respect to 0 azimuthal angle.

The results of embryo alignment are presented in Fig. 2d as snapshots of cylindrical projections of cell coordinates for an embryo at four different stages of development. The coloring vividly shows that the radial component of the spherical coordinates becomes important only after the late gastrula stage. To calculate the cell density, the 3D volume in spherical coordinates was divided into a grid with voxel size of 2π/100 × π/50 × 0.03 for the azimuthal (φ), elevation (θ) and radial (R) axes, respectively, and the number of cells in each voxel was normalized to the total number of cells. We note that the voxel size was chosen such that there are ~4 cells per voxel surface on average (100 × 50 pixels for the entire surface) at 100% epiboly, and the radial value of 0.03 corresponds to ~10 μm, i.e., about half of the hypoblast layer thickness at 7 hpf. In Fig. 2e, the normalized cell densities are shown for the same sample as in Fig. 2d. For each voxel in the grid, we also calculated the cell movement direction at all times between 5 and 12 hpf by averaging the displacement of all cells appearing in the volume of a particular voxel over the time course of 10 min.

### Ensemble-averaged digital embryo model

**Figure 3:**
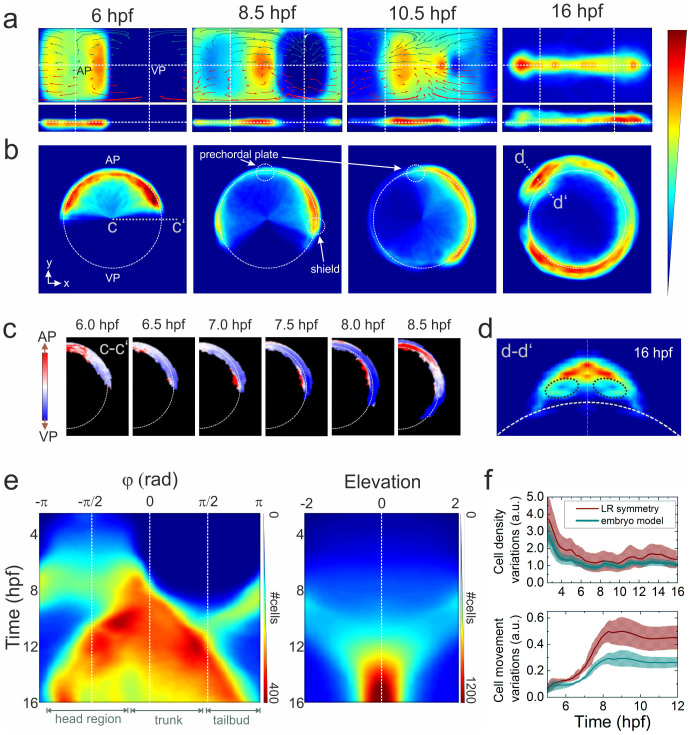
Ensemble-averaged digital model. (a) Averaged cell density cylindrical projections in spherical coordinates (azimuth vs. elevation). The vector fields represent 2 h collective cell migration patterns, computed by locally averaging the displacement of cells with R < 1 (red arrows) and R > 1 (dark cyan). For symmetry reasons, only half of each vector field is shown. (b) Planar projections in Cartesian coordinates at selected times; the dashed white line indicates the embryo sphere, and the blue-to-red color code represents the normalized cell density (in arbitrary units). (c) Projection of cell density movement at the embryo dorsal side from 6 to 8.5 hpf; the direction is encoded by color (toward the AP: red, toward the VP: blue). (d) Cell density projection at 16 hpf at a cross-section through the head region (d–d′) depicted in panel (b); the dashed black lines mark the eye positions. The color code is the same as in (a) and (b). (e) Kymographs of azimuthal (left panel) and elevation (right panel) cell density projections; the color code denotes the number of cells per azimuthal or elevation segment, respectively. (f) Quantitative analysis of cell density (upper panel) and movement (lower panel) variations with respect to the midline mirror (left-right) symmetry (within each sample) and to the model. The solid lines represent averages over the ensemble; the shaded regions around the lines depict the ±σ values of statistical significance that include sample heterogeneity as well as statistical errors due to cell number variations within voxels (σ^2^ = (σ_samples_)^2^ + (〈σ_ΔN_〉)^2^).

After careful alignment of the individual data sets to the standard orientation, the data from all samples were combined to an average cell density on a 4D (3D + t) grid, and cell movement directions were also calculated on a 4D grid. Cell densities and movement directions were additionally averaged with respect to the midline symmetry to account for the expected left-right symmetry along the anteroposterior axis, which holds for normally developing embryos until the 10–12 somite stage (~14–15 hpf), when the left-right patterning of visceral organs begins in Kupffer's vesicle^22^. The resulting 2D maps of cell densities at 6, 8.5, 10.5 and 16 hpf are displayed in Fig. 3a using the Gall-Peters (2 sinθ versus φ) and the R versus φ projections. To visualize collective cell migration from a certain region of embryo over the course of development, we iteratively calculate the relative displacement of each voxel in that region. In Fig. 3a, we display the vector fields of collective cell migration over 2 h from evenly distributed regions (size π/10 × π/10 rad^2^) across the surface. The data were analyzed separately for cells with R < 1 (red arrows) and R > 1 (dark cyan arrows), representing presumed hypoblast and epiblast cells, respectively. Although the analysis is coarser, based on cell density rather than on single cell behavior, these collective cell displacements agree very well with those obtained by tracking individual cells (Supplementary Fig. 4). We can further display the cell density in a more conventional, real-space representation by back-transforming the digital model from spherical to Cartesian coordinates (Fig. 3b).

The analysis of the averaged cell density and displacement over time, as plotted in Fig. 3a and 3b, is a useful tool to visualize characteristic changes in embryo morphogenesis at early developmental stages. Remarkably, all characteristic morphogenetic changes including shield formation (Fig. 3c) or eye development in the head region (Fig. 3d) are clearly reproduced by the model. The three major morphogenetic events occurring during gastrulation, epiboly, convergent extension and internalization, are faithfully reproduced. Epiboly, the movement of cells toward the VP, is accompanied by the spreading and thinning of cell layers. Up to the shield stage at 6 hpf (Fig. 3a), epiboly is observed as a bidirectional movement of cells along the azimuthal axis with a constant migration velocity of ~1 μm/min at all elevation levels, appearing as a lateral elongation of a rectangular region of cells centered on the AP. Because there are no obvious variations in radial position, this plate of cells forms a flat sheet, in spite of the greater cell density along the two edges of the migration front at the germ ring. By the time when epiboly reaches 75% (8.5 hpf), cells spread over the entire surface of the azimuth-elevation field, leaving a circular cell-free space centered on the VP (Fig. 3a). Nuclei from the yolk syncytial layer (YSL) form concentric ring patterns around the VP (Fig. 2d, 8.5 hpf). In contrast to early epiboly, cell density varies considerably along the elevation axis at all azimuthal angles, showing a positive correlation to the radial position of the enveloping layer nuclei. This is most pronounced in the shield, showing the highest cell density and radial positions (Fig. 3b, 8.5 hpf). Similarly, the lower cell density at the AP region is associated with a lower radial position of the enveloping layer nuclei, except for the prechordal plate, which forms an island with slightly larger radial thickness. The progression of the epiboly movement is transformed into a continuous closing motion of the cell-free circle at the VP (100%-epiboly and bud stage, 8.5–10.5 hpf, Fig. 3a). Convergent extension is observed as a narrowing and extension of the body along the anterior-posterior axis (midline). Interestingly, for the time period from 10 to 12 hpf, extension along the azimuthal axis appears as a unidirectional, leftward movement only. This is due to a shift of the center of azimuthal symmetry applied for sample alignment; beyond 12 hpf, extension along the azimuthal axis shows bidirectional movement. The movement of epiblast and hypoblast nuclei during late gastrulation stages (8.5–10.5 hpf) form clockwise and counter-clockwise “swirls”. At later times (10.5 hpf), the centers of these swirls are located near the lateral boundary of the eye field for both the hypoblast and epiblast layers, whereas at earlier times (8.5 hpf), the center of the hypoblast swirl is significantly shifted toward the VP due to internalization of the hypoblast cells and their ensuing migration in the opposite direction of the epiblast cells (Fig. 2c)^23^.

The 2D projection facilitates 3D data viewing at early developmental stages. This visualization, however, is not suitable for showing kinetic data. For observing continuous changes in the cell distribution over time, we generate kymographs, in which either azimuth or elevation is plotted as a function of time of development. To this end, we integrate the data along one of the axes in the density map, thereby collapsing the 2D map into a line; colors encode the absolute number of cells per segment (Fig. 3e). All major morphological changes discussed above can also be identified easily, and the time of the respective process is directly visible.

The quality of the ensemble-based digital embryo model rests on the precision with which individual data sets can be aligned prior to averaging. As a means of assessing this precision, we have analyzed sample-to-sample variations of cell density and movement. The ensemble average of cell density variations in volume segments symmetrical to the elevation midline (left-right symmetry) are plotted in Fig. 3f (upper panel) as a function of time. Also shown are cell density variations between the individual samples and the ensemble-based digital embryo model. For both quantities, variations within individual samples and between individual samples and the model decrease up to 6 hpf because the number of cells and, concomitantly, the local cell density increases. In the same fashion, we have also compared cell movement variations between samples (Fig. 3f, lower panel). The high symmetry of cell movement with respect to the AP at early times (<7 hpf) gives rise to only minute cell movement variations within individual samples and between individual samples and the model. At later times, when the body axis starts to develop, morphogenetic changes and the tilt of individual samples due to the embryo mounting in agarose may produce larger differences. We note that variations between the individual samples and the model are always smaller than the left-right cell density variations within each sample, attesting to the precise overlay of individual samples.

### The digital model as a canvas for embryo morphogenesis

The digital embryo model can serve as a reference frame within which the behavior of defined subpopulations of cells can be studied. Such subpopulations can be selected in various ways, for example, by their specific fluorescence staining or by their positions or dynamics within the embryo.

**Figure 4:**
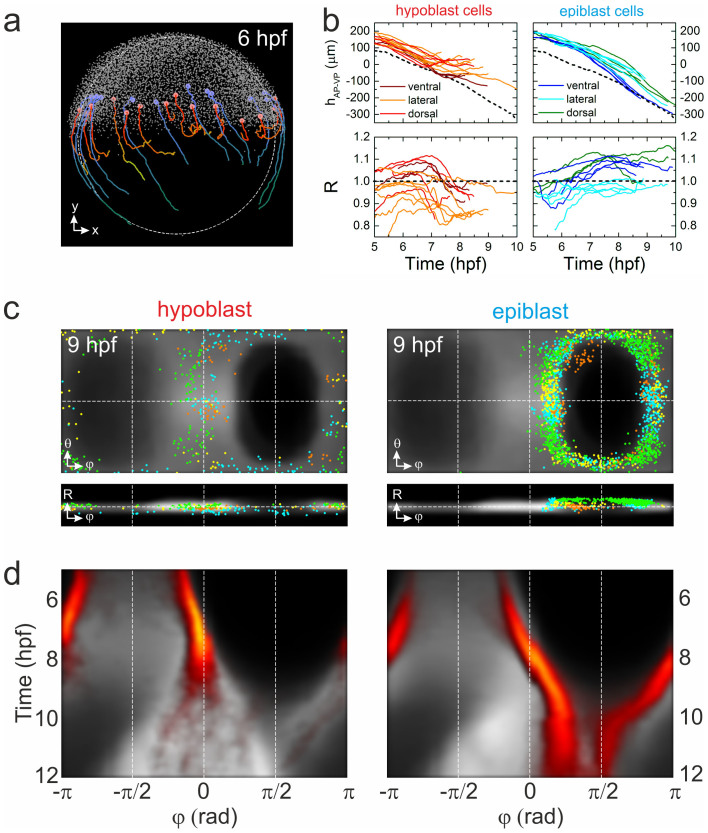
Visualization of blastoderm margin development. (a) Lateral view of a zebrafish embryo at 6 hpf; cells are shown as small gray dots. The colored spheres denote examples of hypoblast (red) and epiblast (blue) cells; also shown are their trajectories from 6–10 hpf. (b) Typical trajectories of selected hypoblast (left) and epiblast (right) cells during gastrulation; represented by their positions projected onto the AP-VP axis (top) and their radial positions (bottom). Trajectories are color-coded according to dorsal, lateral or ventral position; the dashed line (upper graphs) represents the position of the blastoderm margin. (c) Gall-Peters projections and radial plots of cell positions, showing an overlay of hypoblast (left) and epiblast (right) cells from different samples (shown in different colors) on top of the averaged model cell density (gray) at 9 hpf. (d) Hypoblast (left) and epiblast (right) azimuthal cell density kymographs (yellow/red) overlayed onto the digital model kymograph from Fig. 3e (gray).

As an example, we have analyzed cell migration associated with germ layer formation^23^,^24^,^25^. At ~50% epiboly, the margin of the blastoderm thickens to form the germ ring. Mesendo-dermal progenitor cells in the germ ring migrate to the margin, move downwards toward the yolk and, subsequently, back in the direction of the AP (Fig. 2c). They form the hypoblast cell layer, whereas the cells of the surface layer continue to advance toward the VP to form the ectodermal epiblast layer. To visualize typical trajectories of this cell rearrangement for an individual embryo, we have identified all cells appearing within 100 μm of the blastoderm margin during gastrulation and analyzed their trajectories if they exceeded 120 min. Cells with trajectories showing internalization and increasing separation from the margin were assigned to hypoblast cells, whereas those that followed the advancing blastoderm margin (excluding the enveloping and yolk syncytial layer cells) were presumed to be epiblast cells. Using these rather stringent criteria, we assigned ~15% and ~45% of all cells at the blastoderm margin at 6 hpf to hypoblast and epiblast cells, respectively. In Fig. 4a, we show the embryo at 6 hpf. The different migration patterns of hypoblast and epiblast cells, depicted in red and blue, respectively, are obvious from a few representative trajectories (6–10 hpf) shown in the figure; all identified cells and their respective trajectories are shown in Supplementary Fig. 4. The different migration patterns of the cells are also evident from plotting their displacements from the blastoderm margin and their radial positions as a function of time (Fig. 4b).

Hypoblast and epiblast cells from different samples are shown in Gall-Peters and radial plots in Fig. 4c. There, we have overlaid all identified cells with the cell density digital model to visualize the behavior of hypoblast and epiblast cells in the context of the entire embryo. The differences in the patterns are obvious. While the identified hypoblast cells are scattered across the entire cell density, the epiblast cells remain close to the blastoderm margin. A high degree of symmetry is seen with respect to the midline, although the distribution of cells from individual embryos is rather inhomogeneous, presumably due to the stringent cell selection and identification criteria. Nevertheless, ensemble averaging of identified hypoblast and epiblast cells represents their behavior depicted in Fig. 4d in the form of azimuthal kymographs. Thus, the identified hypoblast cells contribute mostly to the formation of the trunk region (φ ≈ 0) at later developmental stages, whereas the epiblast cells continue forward to the VP (φ ≈ π/2).

### Detection of morphological changes of a zebrafish mutant at early developmental stages

**Figure 5:**
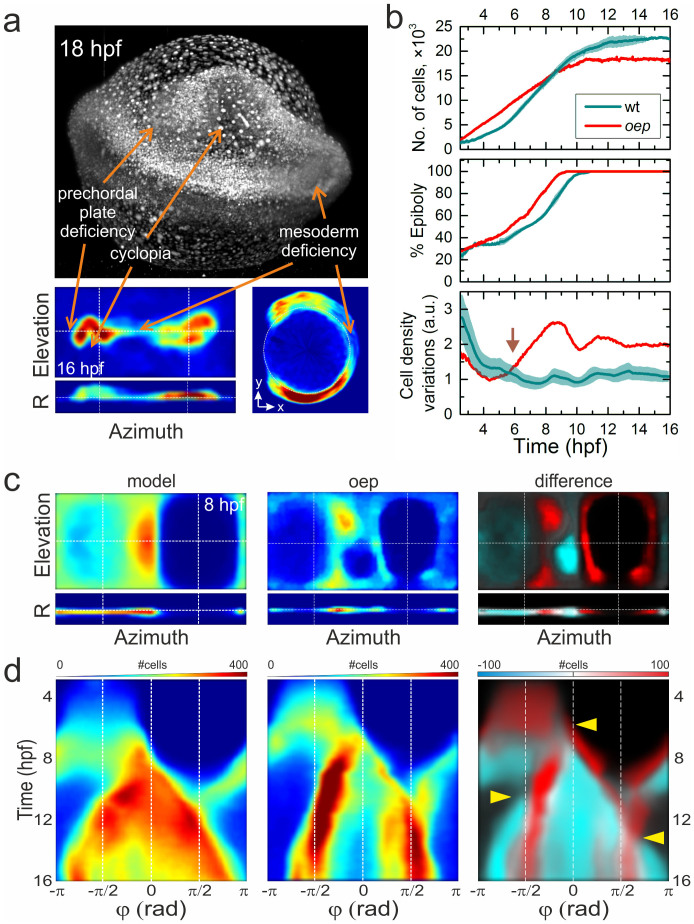
Morphological changes of an *oep* mutant. (a) MIP image of an *oep* mutant embryo at 18 hpf and cell density projection at 16 hpf. Arrows indicate characteristic phenotypical *oep* features. (b) Quantitative comparison of *wt* and *oep* mutant cell number (top), epiboly (middle), and cell density variation during development (bottom). Data of the *wt* embryo (Fig. 3F and Supplementary Fig. 3) are included for comparison. (c) Left and middle panels: Cell density projections at 8 hpf of the averaged model and the individual *oep* mutant, respectively, depicted in the same color code as in Fig. 3a. Right panel: Overlay of differences (negative: cyan, positive: red) in cell densities between the mutant and the *wt* model on top of the model data (gray). (d) Kymographs of azimuthal projections, panels arranged as in (c). Arrows point to pronounced developmental differences between the *oep* mutant and the *wt* embryo model.

To enquire if a comparison with the ensemble-based digital embryo model can uncover abnormal development already at very early stages, where they are not apparent from plain visual inspection, we studied a zebrafish embryo containing the well characterized *oep* mutation, known to cause abnormal forebrain, mesoderm and endoderm formation^26^,^27^,^28^,^29^. The *oep* cyclopia phenotype is clearly visible in the MIP image at 18 hpf or in the 2D projection of cell density at 16 hpf (Fig. 5a). The forebrain, hindbrain and spinal cord regions show significantly reduced cell density. After embryo alignment, the number of cells, epiboly, and cell density variations were calculated as a function of developmental time (Fig. 5b). Notably, the cell proliferation rate and the epiboly development differ significantly already at the early gastrula state. Remarkably, the cell proliferation rate of the *oep* mutant remains essentially constant between 2.5 and 10 hpf, being faster at the blastula stage and smaller at the gastrula stage than the one of the control wild-type (*wt*) embryos. Consequently, the *oep* mutant has a larger number of cells at the blastula and early gastrula stages, but a smaller number of cells at the late gastrula and segmentation stages. Also, in contrast to control embryos, the *oep* mutant does not display a clear pause in proliferation during germ ring and shield formation around 5 hpf. Quantitative comparison between the model and the mutant cell densities clearly reveals statistically significant differences already at 6 hpf (Fig. 5b, lower panel). By comparing 2D cell density projections at 8 hpf (Fig. 5c), differences in cell densities are evident at the entire blastoderm margin and in the shield and AP regions. The propagation of these differences over time is nicely seen in the azimuthal kymographs in Fig. 5d; time points beyond which the morphogenetic defects become detectable are marked with yellow triangles. Thus, in the trunk region, mesoderm deficiency is caused by a significantly reduced germ layer formation at the onset of gastrulation (middle mark (φ ≈ 0) at 6 hpf in Fig. 5d, Supplementary Fig. 6a), the reduced number of cells in the AP region at 8 hpf causes a deficiency at the hatching gland and prechordal plate (left mark at ~10 hpf in Fig. 5d) and the tailbud region (right mark at ~13 hpf, Fig. 5d) at the segmentation stage. To clarify the contribution of these defects to formation of hypoblast and epiblast cell layers separately, we first attempted to identify hypoblast cells by their morphogenesis at the blastoderm margin by using the same criteria as described in the previous section. We found that only ~3% of all cells at the blastoderm margin can be assigned to hypoblast cells, while the fraction of epiblast cells, ~45%, remained the same as for *wt* embryos. All identified cells and their trajectories are shown in Supplementary Fig. 6b. Furthermore, due to our inability to unambiguously identify all cells, we approximated the hypoblast and epiblast cell layers by separately considering cells that appear respectively below or above the sphere of unit radius. By comparing the time development of the number of cells for the *wt* embryo and the *oep* mutant, a clear similarity can be revealed for the “epiblast” layer, at least up to 11 hpf (Supplementary Fig. 6c). Beyond 11 hpf, an anti-correlated increase in the number of “epiblast” cells accompanied by a decrease in the number of “hypoblast” cells for the *wt* embryo is presumably caused by a radial displacement of the germ layers due to cell convergence to the midline and cell density increase. Notably, the number of “epiblast” cells of the *oep* mutant does not show any further increase beyond 11 hpf, but rather stays on the same level reached by the end of gastrulation at ~10 hpf. In contrast to the “epiblast” cells, the “hypoblast” layer of the *oep* mutant shows a two-fold faster proliferation rate than *wt* samples until ~6 hpf, followed by a drastic decrease in the number of cell to about half of the level of *wt* embryos. Furthermore, by comparing cell densities of only the “hypoblast” layer between the *wt* and the *oep* mutant embryos, a clear difference is evident at the AP region (Supplementary Fig. 6d). At 6 hpf, cells are more or less evenly distributed across the animal plate for both the *wt* and the *oep* samples, although the *oep* mutant has a larger cell density. At 8 hpf, however, cells at the AP, which is the region where the presumptive prechordal plate and its derivative hatching gland are formed^18^, are almost absent for the *oep* mutant. Our analysis yields a clear identification of the origin of morphogenetic defects in the *oep* mutant: yet, the detailed molecular mechanisms causing the defects in the formation of the hypoblast cell layer at the onset of gastrulation still remain to be elucidated.

## Discussion

DSLM is an excellent experimental technique for studying the development of model organisms in great detail, including variants with abnormal development. Often, these differences become obvious only at later stages or even remain subtle throughout the entire development process. For enhanced sensitivity, digital models of cell density and movements are required that average over traits of individual samples. Here we have presented a fast DSLM data acquisition platform that allows huge data sets of many terabytes to be taken within a few days. For the zebrafish model system, we have shown that multiple data sets can be precisely aligned and averaged, so that a digital model of cell density and movement of the entire ensemble could be constructed. This digital model may serve as a canvas, on which the behavior of cellular subpopulations can be studied. These subpopulations may be defined by selective labeling with fluorescent markers or by their specific behavior, as exemplified here by the different migration patterns of hypoblast and epiblast cells. Furthermore, the model can be utilized to visualize coherent cell migration in particular regions by computing a collective cell displacement vector field. For example, morphogenetic changes of the prechordal plate − a region of hypoblast cells at the AP^18^ − can be visualized as a time series of its cell density projection, with the highlighted region of cells selected at a specific time point (Supplementary Fig. 7a). However, limitations of our cell density-based digital embryo model become apparent when trying to model very small regions where cells of different fate are in close proximity. A most prominent example of that type is the formation of notochord and somatic mesoderm during gastrulation. While the model is definitely capable of reproducing the convergence and extension cell movements (Supplementary Fig. 7b), it does not distinguish between the notochord and the adjacent adaxial and somite cells that are arranged within a region of only a few tens of microns around the midline^30^. This is a direct consequence of the cell density representation, with a voxel resolution of ~20 μm, which understandably cannot compete with a single cell-based representation. However, while a comprehensive single cell trajectory and cell division analysis from raw multi-terabyte 4D microscopy data is presently still out of reach, we have shown here that our digital embryo model permits a fast, semi-automatized identification of times and loci of phenotypical changes.

The enhanced sensitivity due to ensemble averaging, as shown by comparing the *oep* mutant with the *wt* ensemble, encourages us to apply this strategy to more subtle morphological perturbations in other zebrafish mutants or embryos exposed to altered environmental conditions. Moreover, we note that results from different experiments can be incorporated into the digital model, which greatly facilitates correlating signaling activity with cell position, movement, and lineage. Finally, the approach presented in this work can be applied to different animal species, i.e., other teleosts or even chick, frog and mouse, as long as the developing embryo can be described as an object with a relatively simple shape such as a sphere or an ellipsoid, sample-to-sample size varies significantly less than the observed morphogenetic changes within each species, and single cell-resolved data are available. The first two conditions are fulfilled at the gastrula stage of the above mentioned species^31^, whereas the extension of whole embryo imaging with single cell resolution to more species is still at its beginning^32^,^33^.

## Methods

### Sample preparation

Zebrafish (*Danio rerio*) AB_2_O_2_ wildtype strain, Tg(h2afva:h2afva-GFP) and Tg(tdgf1m134/m134) lines were used in this study. Fish were maintained at 28°C as previously described^34^. Fertilized eggs were obtained upon overnight crossing of adult zebrafish containing the transgenic H2A-GFP fusion protein to render the cell nuclei fluorescent^35^. Chorions surrounding the embryos were removed to reduce background fluorescence, which facilitates cell segmentation and tracking. The zebrafish embryos were mounted in vertically oriented fluorinated ethylene propylene (FEP) tubes, sitting on a plug of 1.5% agarose and being surrounded by 0.1% agarose to minimize effects due to mechanical hindrance (Supplementary Fig. 1c)^36^. Then, they were positioned in the sample chamber filled with fish water medium at 26.5°C.

### DSLM setup

Imaging experiments were performed on a home-built device (Supplementary Fig. 1). In its design, we thoroughly inspected and – wherever possible – parallelized the entire data acquisition process to achieve the highest speed of continuous image acquisition from a volume of ~1 mm^3^ (Supplementary Figs. 1 and 2). Acquisition of a complete 3D image (two opposite views of 1000 μm in depth with 500 frames each) takes about 50 s, corresponding to an effective rate of 1.1 × 10^8^ voxels/s. Acquisition of a single image stack can be further accelerated by adjusting the frame size to only slightly exceed the sample size, and by limiting the recording depth to the part that contributes to the fusion of opposite views. So, by recording only 65% of the entire stack, i.e., 325 frames, the acquisition time is reduced to 16.5 s for a single view or 33 s for a complete 3D image, which is comparable to the acquisition time of 20–30 s achieved by simultaneous multi-view imaging with two detection objectives and two cameras^8^,^9^,^15^.

### Image acquisition and data processing

3D DSLM image stacks were taken continuously for 16 h, starting at the 8–64 cell stage, using laser excitation at 488 nm for GFP and 561 nm for mCherry. The fluorescence emission was collected with a water dipping objective (CFI-75 LWD 16×/0.8w, Nikon GmbH, Düsseldorf, Germany) and detected by an sCMOS camera (Neo, Andor, Belfast, UK). To enable DSLM imaging of zebrafish embryo development with parallel processing of the images, raw data (typically ~10 TB for 16 h of image recording; saved in a multi-page BigTIFF format including an image description tag according to the open microscopy environment (OME) metadata scheme^37^) from the local hard drives were transferred from the microscopy lab to the data storage facility^38^,^39^,^40^ within 8 h and subsequently processed on a supercomputer cluster during the ensuing 24 h using fast cell nuclei segmentation and tracking algorithms^41^,^42^. Superior time-efficiency of the segmentation algorithm is achieved, firstly, by parallel processing of each blobs with detected seed points, and, secondly, by approximating each nuclei as a roundish object using a pixel-weighted mask with the predefined smoothing kernel (for details, see Supplementary Materials and Methods).

### Synchronization of data sets

To synchronize the developmental time axis for different samples, we selected the 256-cell stage as the starting point, corresponding to 2.5 hpf. At this stage, fluorescently marked histone 2A proteins (H2A-GFP) have accumulated in the cell nuclei^35^, so that fluorescence from the nucleus predominates the one from the cytoplasm, which facilitates segmentation of cell nuclei. To match the time of developmental stages at later points with Kimmel's description given for a temperature of 28.5°C, we scaled the measurement time down by 10% because we performed the reported experiments at 26.5°C in the sample chamber.

### Ethics statement

All zebrafish husbandry and experimental procedures were performed in accordance with the German animal protection regulations and were approved by the Regierungspräsidium Karlsruhe, Germany (Az. 35-9185.64).

## Supplementary Material

Supplementary Information Supplementary information

Supplementary Information Supplementary Movie 1
