# Supplementary material for: An ensemble-averaged, cell density-based digital model of zebrafish embryo development derived from light-sheet microscopy data with single-cell resolution
Source: Sci Rep. 2015 Feb 25;5:8601. doi: 10.1038/srep08601 (PMC5390106; doi:10.1038/srep08601)
Supplement: Supplementary Information [file srep08601-s1.pdf]

An ensemble-averaged, cell density-based digital model of zebrafish embryo development derived from light-sheet microscopy data with single-cell resolution

Andrei Yu. Kobitski,[1,2,3] Jens C. Otte,[2,3] Masanari Takamiya,[2,3] Benjamin Schäfer,[1] Jonas Mertes,[1] Johannes Stegmaier,[4] Sepand Rastegar,[2,3] Francesca Rindone,[5] Volker Hartmann,[5] Rainer Stotzka,[5] Ariel García,[6] Jos van Wezel,[7] Ralf Mikut,[4] Uwe Strähle,[3] and G. Ulrich Nienhaus[1,2,3,7,*]

Supplementary materials and methods

Digital scanned laser light sheet microscopy (DSLM) measurements were performed on a home-built setup (Fig. S1a and S1b). It consists of three main components, namely, (i) the optical platform, which includes excitation and detection optical elements and devices, (ii) the sample positioning platform, and (iii) the real-time electronic control and data acquisition platform.

Optical platform. Samples are excited by light from solid-state cw lasers (Omicron LuxX 488 nm and Cobolt Jive 561 nm, Omicron Laserage Laserprodukte GmbH, Rodgau-Dudenhofen, Germany). After passing through a beam expander (AC254-

030-A and AC254-060-A, Thorlabs, Newton, USA) the light beam is guided by a vertically deflecting galvo mirror scanner (8300K, Cambridge Technology, Bedford, USA) to an f-theta lens (S4LFT4375, Sill Optics, Wendelstein, Germany). To enable sample illumination from two opposite sides, the excitation beam is split by a 50/50 beamsplitter (NT49-004, Edmund Optics, Barrington, USA) into two identical illumination arms containing tube lenses and 5×/0.15 air objectives (Nikon, Tokio, Japan). For alignment of the deflected beam (upper illumination path in Fig. S1A), the beamsplitter is positioned on a translational stage (PT1, Thorlabs, Newton, USA) so that it can be moved along the straight beam optical axis (bottom illumination arm in Fig. S1a). For adjustment of the excitation beam waist to specific positions along the optical axis through the specimen, the illumination objectives are placed on translational stages (CT1, Thorlabs, Newton, USA) attached to a common cage system. Homogeneous light sheet illumination is achieved by a linear galvo mirror displacement over the field of view at the rate of 450 Hz. Consequently, for a typical frame acquisition over 40 ms, the light beam scans across the sample more than 35 times, which helps to avoid artefacts related to discrete galvo scanner displacement or camera rolling shutter mode.

The detection unit consists of a 16×/0.8w water dipping objective (Nikon), bandpass filter (Semrock BrightLine HC 525/50 and Semrock BrightLine HC 641/75, AHF, Tübingen, Germany), tube lens (Nikon), and sCMOS camera (Neo, Andor,

Belfast, Northern Ireland). With the camera chip size of 16.6 $\times$ 14 μm^2 and the 16$\times$ magnification of the detection objective, the entire field of view is 1038 $\times$ 875 μm^2, with a pixel size of 0.4 $\times$ 0.4 μm^2, which fits well to the size of zebrafish embryos (up to 750 μm in diameter). Precise alignment of the focal plane of the detection objective with respect to the illumination light sheet is achieved by placing the objective with the firmly attached sample chamber on a linear stage equipped with a differential micrometer (M-462-X-SD and DM-13L, Newport, Irvine, USA).

XYZR positioning platform. The sample positioning platform consists of a motorized vertical translational stage with 4.5 mm travel range and 3.4 μm precision (UZS80CC, Newport, Darmstadt, Germany), an XY piezo stage with 25 mm $\times$ 25 mm travel range and 200 nm precision (PILine M-686, Physik Instrumente, Karlsruhe, Germany), and a rotor stage with sub-milliradian precision (SR-2112-S, SmarAct, Oldenburg, Germany). Additionally, for alignment of the embryo position with respect to the rotational axis, a manual XY translation stage (SCP05, Thorlabs, Newton, USA) is built on top of the rotation stage. Of note, accurate alignment of the rotational axis prior to long-term experiments precludes embryo displacement due to centrifugal forces during rotation. For better mechanical stability of the positioning system, the heavy vertical and XY translational stages are attached directly to the optical table underneath the

sample chamber, whereas the low-weight rotation stage with the sample holder is lifted up by a few centimetres, providing space for the sample chamber below. Opening on top of the sample chamber enables fast and easy sample replacement. To synchronize XY stage movement with image acquisition during 3D stack recording, the translational stage is pre-programmed before the start of the measurement to perform the desired frame-to-frame displacement. Subsequently, program execution is triggered for each frame capture cycle by the camera fire output.

Sample mounting. Fluorescence microscopy measurements were performed on fertilized eggs obtained by overnight crossing of adult zebrafish Tg(h2afva:h2afva-GFP) and Tg(tdgf1m134/m134) lines containing the transgenic H2A-GFP fusion protein. Embryo mounting on our DSLM setup was adopted for optimal performance in the application to very early developmental stages of zebrafish (Fig. 1c). Thus, short fluorinated ethylene propylene (FEP) tubes (BOLA-tube, outer/inner diameters: 1.6/0.8 mm, Bohlender GmbH, Grünsfeld, Germany or Thomafluid-High-Tech-tube, outer/inner diameters: 1.5/1.1 mm, Reichelt Chemietechnik GmbH, Heidelberg, Germany) were first flushed with 0.1 mg/ml BSA solution to prevent attachment of embryos to the tube walls. Then, a small amount of gel solution containing 1.5% low melting agarose (Sigma-Aldrich, München, Germany) with sparsely distributed 100-nm yellow-green fluorescent

beads (Invitrogen, Carlsbad, USA) was soaked into the FEP tube and kept for a few minutes at room temperature to allow the agarose to harden. Afterwards, embryos with manually removed chorion were gently embedded into a solution containing 0.1% low melting agarose and soaked into the remaining part of the FEP tube so that the 1.5% agarose plug was underneath the embryo. Finally, the FEP tube was attached to the sample holder and placed inside the sample chamber. The sample chamber is a diver's helmet assembly of ~15 ml volume made from a stainless steel hollow cube. It has a quadratic cross section; three of its four openings are closed by sapphire windows, the fourth one contains the detection objective. To maintain a constant temperature of typically 26°C inside the sample chamber during the experiments, a heating foil (HK5590, Telemeter Electronic, Donauwörth, Germany) is attached to the bottom of the chamber and connected to a temperature controller (TC200, Thorlabs, Newton, USA). The temperature is monitored by a Pt100 resistance thermometer inserted into the sample medium inside the sample chamber and connected to the temperature controller.

Despite very careful embryo mounting, the animal-to-vegetal pole axis was often tilted from the normal to the surface of the 1.5% agarose layer supporting the embryo. As cells migrate toward the vegetal pole, their movement becomes slowed once they encounter the 1.5% agarose surface. The cells located in the tilt direction experience the frictional effect first, whereas cells on the opposite side

are still exposed to 0.1% agarose solution for some time until they reach the bottom. This asymmetry in cell movement shifts the pole axis toward the point at which the embryo touches the agarose plug.

Real-time electronics and data acquisition system. To control all electronic components and automatically acquire data, we have developed custom C++ based software running under the Windows 7 64-bit operation system (MSVC 2010, Microsoft, Redmond, USA). The software is executed on a workstation assembled by Tooltec (Karlsruhe, Germany) from a Supermicro X8DTG-QF mainboard, 2× Intel Xeon E5620 CPU (16× CPU cores), 96 GB RAM, Areca ARC-1880ix-12 SATA RAID controller, 2× 240 GB OCZ VTX3-25SAT3 solid state drives, 9× 3 TB Hitachi Ultrastar A7K3000 hard disk drives, 2× NVIDIA GeForce GTX 590 graphic cards (4× GPU cores), QLOGIC QLE8240-SR-CK 10Gb network adapter, and a National Instruments NI PCI-6733 multifunction card. To achieve high data spooling rates, all 9 HDDs are joined in a RAID array of level 6 with 21 TB of local disk storage space. The measurement software initializes all electronic devices and sets the acquisition parameters, and it then retrieves images from the camera buffer and stores them locally. All frames from a single 3D stack are recorded in a multi-page BigTIFF format including an image description tag according to the open microscopy environment (OME) metadata scheme. The use of this 64-bit

TIFF file format became necessary because of the file sizes exceeding 4 GB when acquiring typical image stacks of 500 full-sized frames.

Microscope setup performance. The performance of the microscope was evaluated by measuring 40 nm orange fluorescent beads (Invitrogen, Carlsbad, USA) embedded in 1.5% agarose. We note that, by applying bi-directional illumination, we not only improved the homogeneity of sample illumination, but also achieved a minimal average width of the light sheet by displacing the foci of the two excitation beams apart from each other along the optical axis. Thus, by expanding the width of the laser beam to 2.5 mm and by shifting the foci by 100 μm from the centre, the average thickness of the light sheet over the field of view of 800 μm was reduced by ~20% in comparison with single beam illumination focused in the centre. The lateral and axial full widths at half maximum (FWHM) of the point spread function (PSF) are (1.5 ± 0.3) μm and (6.6 ± 1.4) μm, respectively. Of note, the simultaneous bi-directional illumination induces some scattering on the opposite (with respect to the excitation) side of the sample due to beam divergence. Foci displacement, however, also helps to reduce this effect, specifically for zebrafish embryos during early stages of development, when a thin cell layer covers the yolk sphere. Furthermore, we have tested the stability of the microscope by monitoring positions of fluorescent beads over many hours under conditions simulating measurements with zebrafish embryos. The axial and lateral

displacements of the bead positions over 12 h were less than (1.2 ± 0.3) µm and (3.5 ± 0.5) µm, respectively, i.e., less than the optical resolution of the microscope.

Data management. Long-term DSLM data storage is supplied by the Large Scale Data Facility (LSDF) at the Steinbuch Centre for Computing (SCC) at the Karlsruhe Institute of Technology (KIT), which provides an excellent infrastructure with a huge storage capacity (>300 TB for the experiments reported here and >6 PB in total), secure backup of the data in a tape library, fast and parallel access to big data sets and computing facilities. Convenient data handling on the LSDF is assured by the DataBrowser software, which extracts the OME data from each uploaded file and added this information to a metadata database. Moreover, the software provides the possibility to perform standardized image processing, e.g., multi-view fusion, maximum intensity projection (MIP) video compilation or cell nuclei segmentation, directly on the data stored at the LSDF by utilizing a distributed computing environment based on the Apache™ Hadoop® cluster. The cluster consists of 25 nodes, each of them equipped with 2 quad-core Intel Xeon E5520 CPUs @ 2.27GHz and 36 GB of memory. All methods are implemented in custom C++ software using the Insight Toolkit SDK (www.itk.org).

Image processing. Cell nuclei segmentation was performed on each 3D image stack individually. Seed points are detected by finding local extrema in the 26-neighborhood of each pixel on a Laplacian-of-Gaussian (LoG) space-scale

maximum projection, which was iteratively calculated using LoG filtered images. For every detected seed point, a cuboid with side lengths proportional to the radius of the respective blob is cropped out and processed independently, effectively utilizing parallelization to speed up the entire segmentation algorithm. Furthermore, aiming just for the fast approximate segmentation of roundish objects, cropped regions are treated by a simple pixel weighting mask based on the dot product of the seed point normal (a vector pointing away from the seed point) with the corresponding normalized gradient vector at that pixel and the distance from the pixel to the seed point. This procedure yields a transformed image that can be segmented by a straightforward Otsu's threshold selection method. The developed algorithm resulted in an outstanding performance in terms of time per data size, still providing sufficient segmentation quality for basic statistical analysis of cell populations. Thus, a data set of 10 TB can be processed in less than 12 h.

Segmentation of fused multi-view images results in artefacts in the middle of image stacks (frame index 200 – 300) due to the time shift of 25 s between two consecutive views, as was shown by Keller et al. for *Drosophila* embryos. These artefacts have been reduced by segmenting the raw data of each view separately, and merging the data that were corrected for cell duplication. To reduce the amount of false positive detections, we used several features implemented in the

segmentation algorithm and manually determined trapezoidal fuzzy set membership functions on the basis of feature histograms for each feature. This allowed us to calculate fuzzy membership degree values for each object in a range between 0 and 1, corresponding to its validity regarding a selected feature. The most discriminative features turned out to be the object volume, the ratio of foreground vs. background mean intensity, the ratio of foreground vs. background volume and the length of the minor axis of the object. Additionally, object membership values were weighted according to their respective distance to the detection objective, to reduce the amount of erroneous detections in heavily blurred image regions opposed to the detection objective. The membership values of every detected segment were combined by multiplication and, subsequently, objects with a combined membership value of less than 0.5 were discarded. Based on visual inspection of the maximum intensity projection, this approach worked well in practice. Furthermore, redundant objects detected in both complementary views were fused if their distance was smaller than the minor axis. Objects were combined by a weighted sum of the feature vectors. The weighting was performed according to the distance from the detection objective, i.e., closer objects contributed more to a fused object, to preserve feature quality of objects that were detected in sharp image regions while reducing the weight of nuclei that were detected in low quality image regions.

To reveal the migration pattern of individual cells during gastrulation, temporal correspondences of the detected nuclei have been identified using a nearest neighbor tracking algorithm implemented in the Matlab toolbox Gait-CAD. During the first iteration, the maximum displacement speed of associated objects between two time points was set to 0.2 µm/s; objects missing in a single frame and reappearing in a subsequent one were assigned to the same trajectory. With this procedure, we achieved an average frame-by-frame association rate of 89.1% including initial time points where nuclei detection was hardly possible. In the second iteration, incomplete tracks were fused over a larger time span using an additional nearest neighbour approach to connect start and end positions of the identified tracks in the spatio-temporal domain. The maximum temporal distance was constrained to ±300 s, with a permitted displacement speed of 0.17 µm/s based on the average nucleus movement. We note that complete lineage reconstruction at the single cell level was not possible over the entire time interval; still, trajectories were revealed spanning several hours.

Supplementary figures

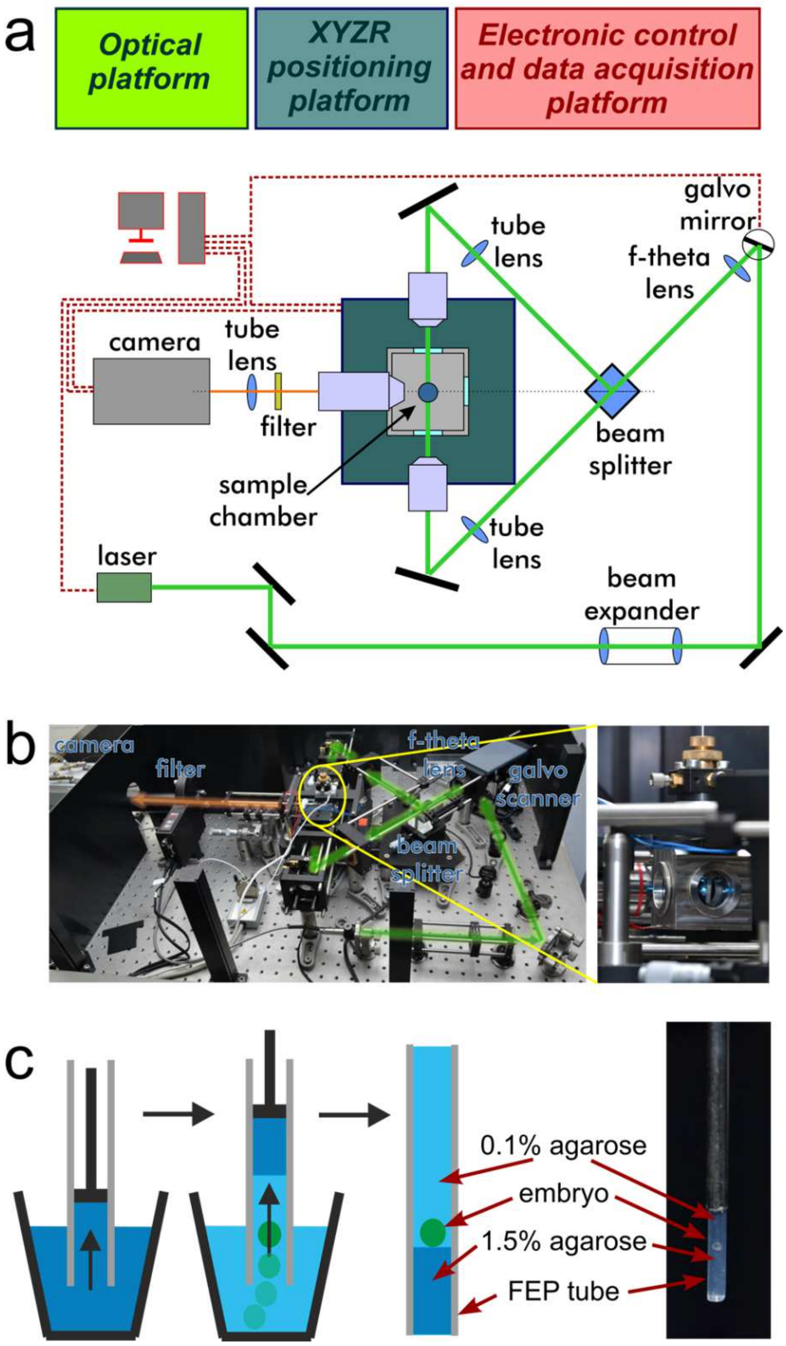

Supplementary Figure 1. DSLM experimental setup. **(A)** Schematic representation of the microscopy apparatus consisting of optical, sample positioning, and electronic control and data acquisition platforms. **(B)** Photographs of the experimental setup. *Left:* Overview; green and orange arrows depict the excitation and detection light paths, respectively. *Right:* Close-up of the sample chamber. **(C)** *Left:* Zebrafish embryo mounting procedure. *Right:* Photograph of the sample holder with a zebrafish embryo embedded in 0.1% low melting agarose inside the FEP tube.

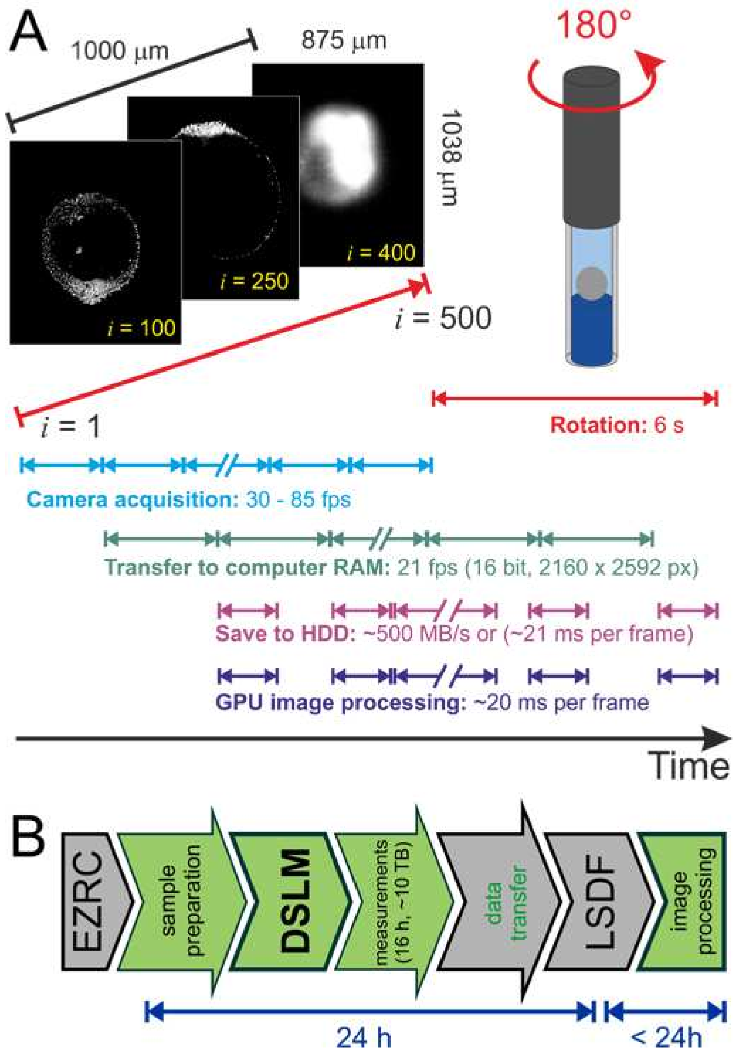

Supplementary Figure 2. (a) Schematic of the process flow during DSLM measurements of zebrafish embryo development. One cycle consists of a 3D stack acquisition followed by a sample rotation by 180°. The camera acquisition rate is set as high as necessary to finish one cycle within the time of complete data transfer from camera to computer. **(b)** High-content framework for zebrafish development experiments. Adult fishes maintained at the European Zebrafish

Resource Center (EZRC) are crossed in the morning prior experiments. Obtained embryos are pre-screened for the fluorescence signal and transported to the microscopy lab. Sample mounting, 16 h time-lapse 3D image recording and data transfer to Large Storage Data Facility (LSDF) are completed within 24 h. Standard image processing such as cell nuclei segmentation and tracking is completed within less than 24 h after the measurements.

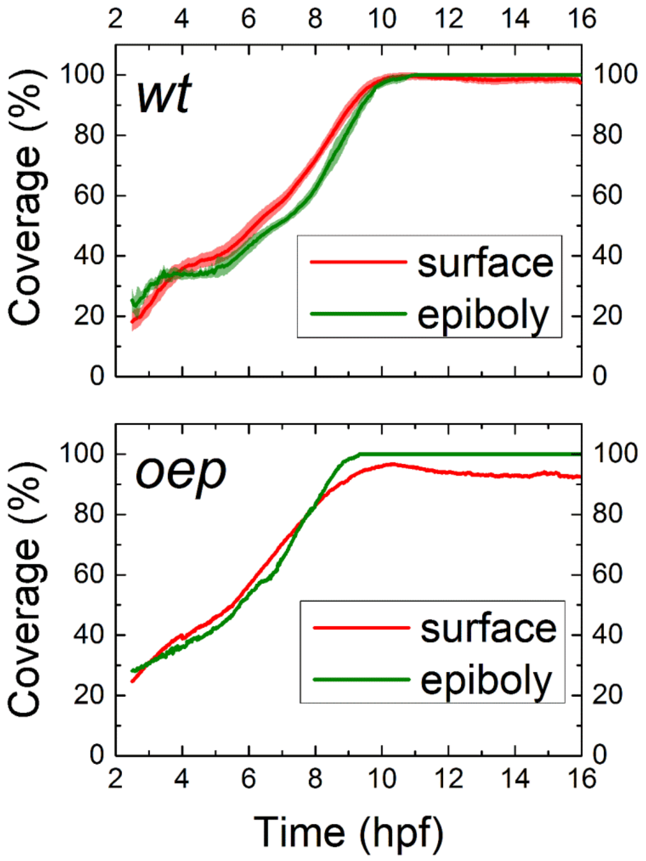

Supplementary Figure 3. Comparison of surface coverage and epiboly time-dependence during embryo development for the *wt* (upper panel) and *oep* mutant (lower panel) embryos.

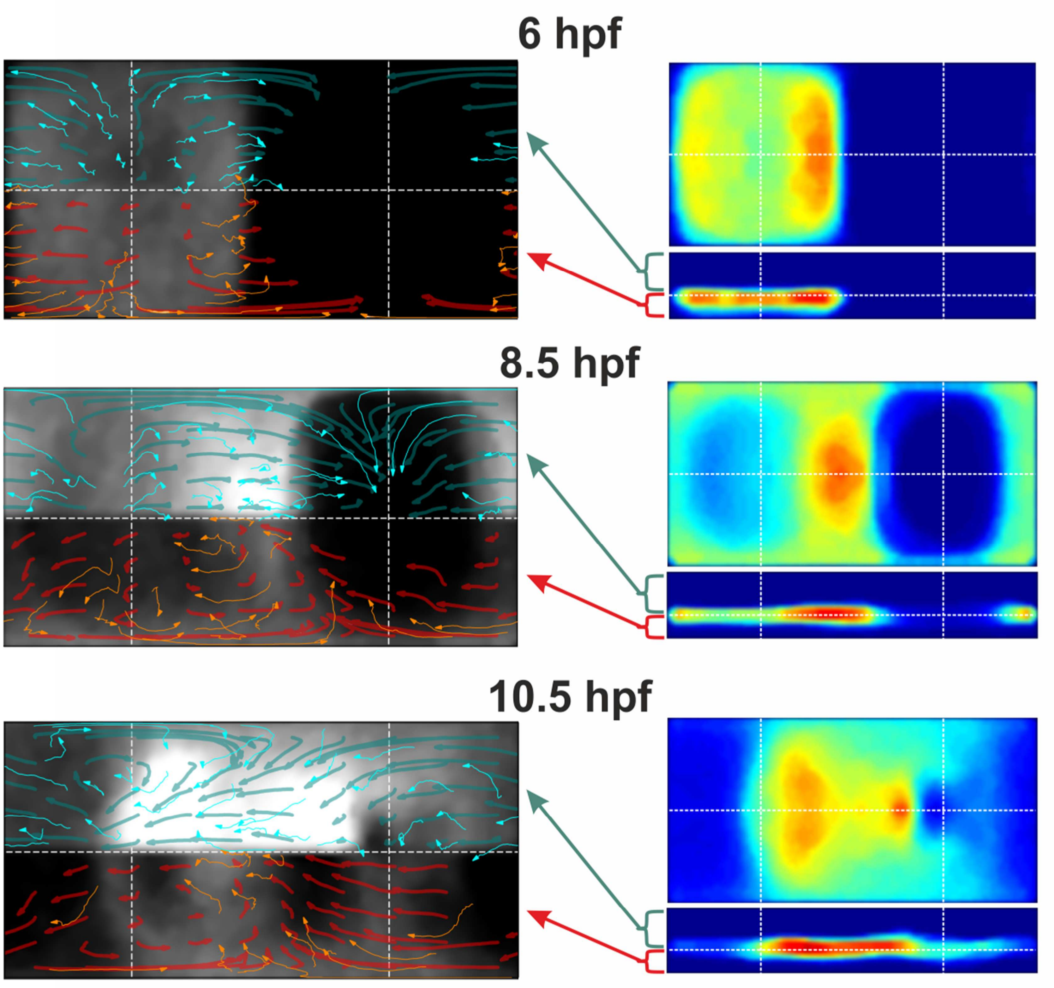

Supplementary Figure 4. Comparison of densities (grey map) and two-hour collective migration pattern (arrows; same as in Fig. 3a in the main text) of "epiblast" (upper half of the cell density maps and dark cyan arrows) and "hypoblast" (lower half of the cell density maps and red arrows) cell layers Additionally, individual two-hour cell tracks are shown for each type of cell layers.

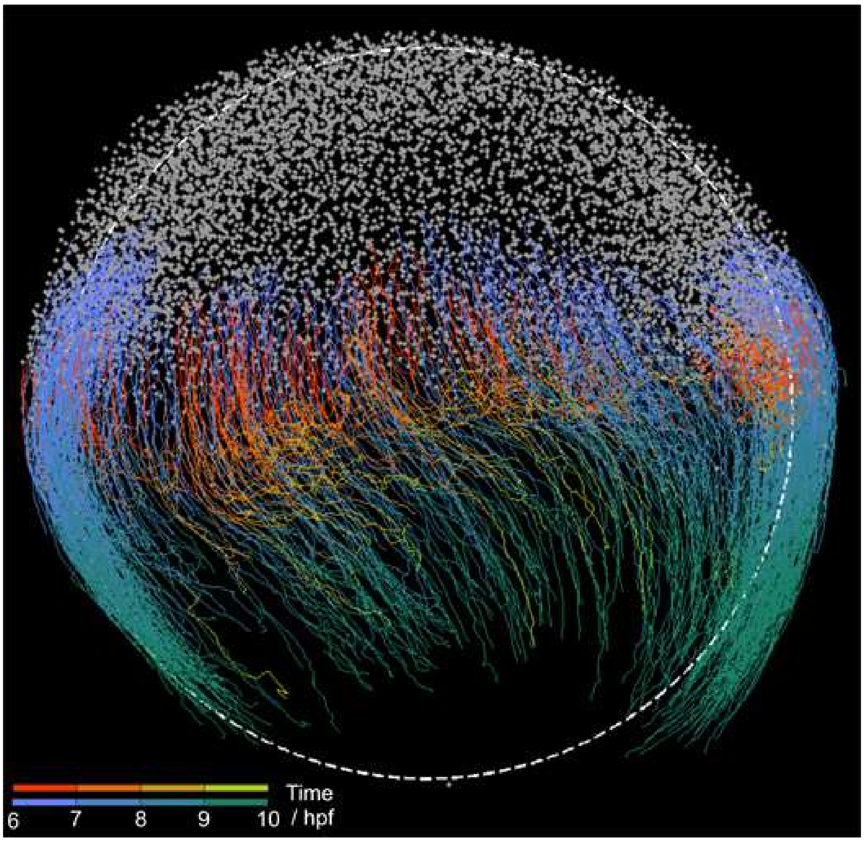

Supplementary Figure 5. Lateral view of zebrafish embryo at 6 hpf; cells are shown as small gray dots. Trajectories from 6 – 10 hpf of hypoblast (red/yellow, n = 245) and epiblast (blue/green, n = 859) cells are show as colored lines.

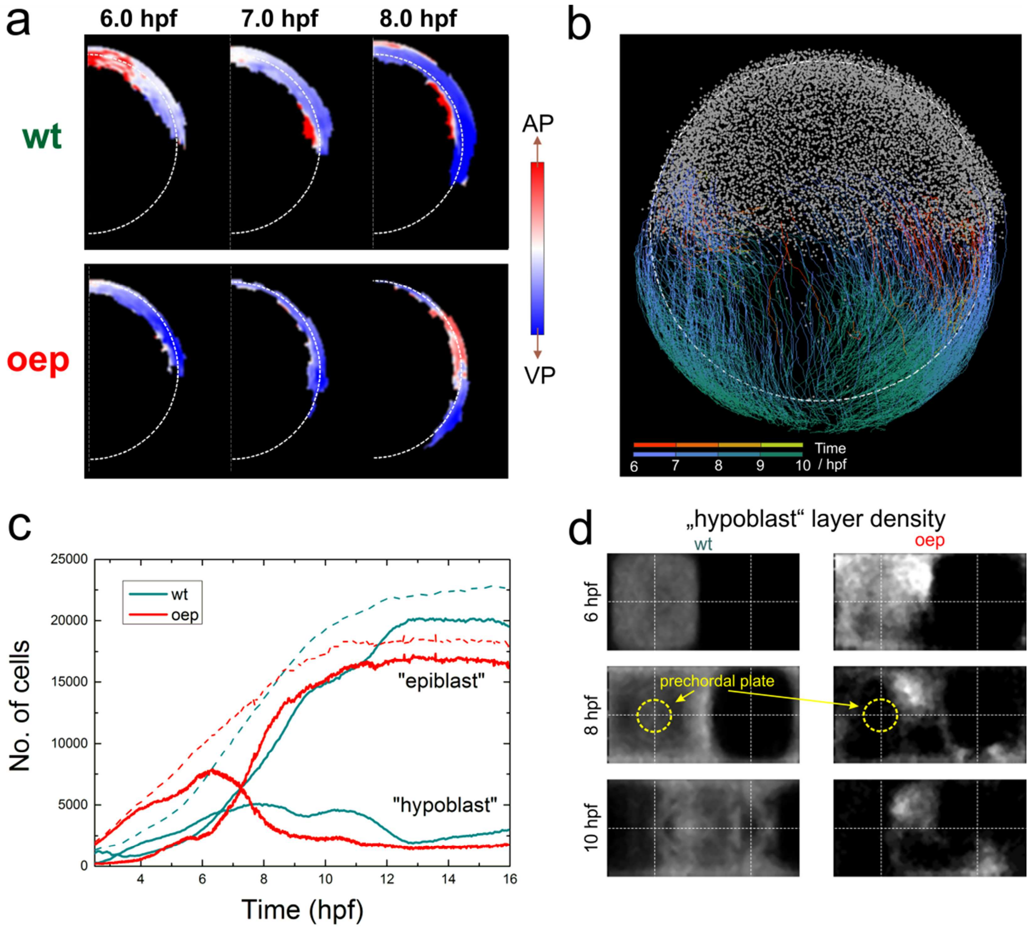

Supplementary Figure 6. (a) Projection of cell density movement at the embryo dorsal side from 6 – 8 hpf for *wt* (top) and *oep* mutant (bottom) embryos; the migration direction is encoded by color (toward the AP: red, toward the VP: blue, as in Fig. 3c). **(b)** Lateral view of zebrafish *oep* mutant embryo at 6 hpf, with cells shown as small gray dots. Trajectories from 6 – 10 hpf of hypoblast (red/yellow, n = 74) and epiblast (blue/green, n = 1278) cells shown as colored lines. **(c)** Time dependence of the total number of cells with the radial coordinate R > 1 (“epiblast”) and R < 1 (“hypoblast”) cells for the *wt* and *oep* samples (solid lines);

dashed lines depict the number of all cells of each sample as in Fig. 5b, top panel. **(d)** Cell density of the “hypoblast”-like cell layer at selected time points of 6, 8 and 10 hpf. Dashed circles depict the appearance and the absence of the presumptive prechordal plate for the *wt* (left) and the *oep* mutant (right) embryos, respectively.

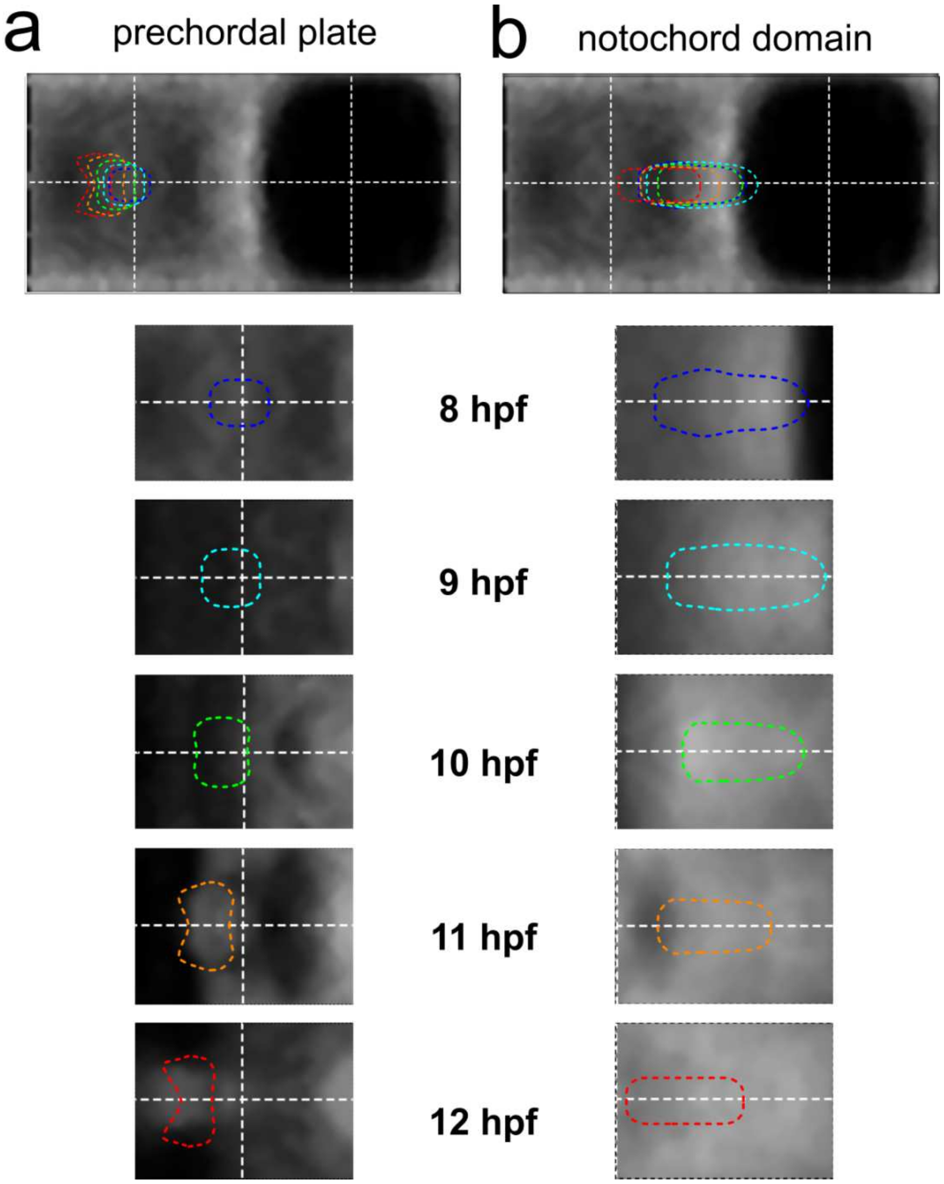

Supplementary Figure 7. Migration patterns of **(a)** prechordal plate and **(b)** notochord domain, depicted as cell density regions marked by colored dashed lines at 8, 9, 10, 11 and 12 hpf (top panels). The smaller panels are close-ups of the regions marked in the top panels.

Supplementary Table 1. Structure of the Matlab file (version 7.3) that contains the ensemble-averaged digital embryo model (available at http://www.aph.kit.edu/nienhaus/english/26_409.php#DigitalEmbryoModel).

Data variables		Variable Dimensions	Description
**model_info**		1	Structure variable with general information on the model
	NSmpl	1	Number of averaged samples
	BinsAzim	1 × 101	Azimuthal (φ) axis scale
	BinsElevDens	1 × 51	Elevation axis scale in Gall-Peters projection
	BinsElevDisp	1 × 51	Elevation (θ) axis scale in equidistant projection
	BinsDist	1 × 30	Radial (R) axis scale
	DisplacementTime	1	Time period for cell movement direction averaging
**model_data**		270	Structure array with the digital embryo data for every time point
	time_hpf	1	Developmental time in hours
	NoCells	1	Averaged number of segmented cells per sample
	NoCellsVar	1	Standard deviation of the number of cells from sample averaging
	Coverage	1	Average surface coverage (in percent)
	CoverageVar	1	Standard deviation of the surface coverage from sample averaging
	Epiboly	1	Average epiboly development (in percent)
	EpibolyVar	1	Standard deviation of

			epiboly development from sample averaging
	Density	101 × 51 × 30	3D grid array of average cell number per voxel normalized to the total number of cells (φ × θ × R)
	CellSum	101 × 51 × 30	3D grid array of cell number per voxel summed over all samples (φ × θ × R)
	Displacement	101 × 51 × 30 × 3	4D vector array of average cell movement directions per voxel (φ × θ ×R × (Δφ,Δθ,ΔR))
	KymoAzim	1 × 101	Array of cell density data summed along the radial and elevation axes, multiplied by the total number of cells
	KymoElev	1 × 51	Array of cell density data summed along the radial and azimuthal axes, multiplied by the total number of cells

Supplementary Files

Supplementary Movie 1. DSLM time-lapse video of MIP images of a zebrafish early stage embryo development.
